# Microbiological findings in a cohort of patients with coronavirus disease 2019 and venovenous extracorporeal membrane oxygenation

**DOI:** 10.1007/s00063-024-01245-6

**Published:** 2025-01-31

**Authors:** Christian Glück, Eugen Widmeier, Sven Maier, Dawid L. Staudacher, Tobias Wengenmayer, Alexander Supady

**Affiliations:** 1 Interdisciplinary Medical Intensive Care, Medical Center—University of Freiburg, Faculty of Medicine, University of Freiburg, Hugstetter Straße 55, 79106 Freiburg, Germany; 2 Department of Cardiovascular Surgery, Heart Center, University of Freiburg, Freiburg, Germany

**Keywords:** Acute respiratory failure, *Staphylococcus spp.*, COVID-19, *Candida spp.*, *Klebsiella spp.*, Akutes Atemnotsyndrom, *Staphylococcus spp*., COVID-19, *Candida spp.*, *Klebsiella spp.*

## Abstract

**Background:**

Venovenous extracorporeal membrane oxygenation (VV ECMO) is an established support option for patients with very severe respiratory failure and played an important role during the coronavirus disease 2019 (COVID-19) pandemic. Bacteria and fungi can lead to severe infectious complications in critically ill patients. The aim of this study was to describe the microbiological spectrum of bacteria and fungi detected in patients with COVID-19-associated respiratory failure supported with VV ECMO in our center.

**Methods:**

This retrospective single-center analysis included all patients with COVID-19-associated respiratory failure supported with VV ECMO in our center between March 2020 and May 2022. All findings from microbiological samples, taken as part of clinical routine assessment from initiation of VV ECMO until day 30 were included. Samples were described by site and time of detection and microbiological characteristics.

**Results:**

From March 2020 through May 2022, 88 patients with COVID-19-associated respiratory failure received VV ECMO support at our center. In 83/88 patients (94.3%), one or more pathogens were found in microbiological samples. Most pathogens were isolated from samples from the respiratory tract (88.6%). Earliest detection occurred in samples from the respiratory tract with a median time of 5 days to first detection. The most frequently detected pathogens were *Staphylococcus spp*., *Candida spp., Klebsiella spp., Escherichia coli* and *Enterococcus spp.*

**Conclusion:**

In this cohort of severely ill COVID-19 patients receiving VV ECMO support, pathogens were frequently detected.

## Abstract (de)

**Hintergrund:**

Die venovenöse extrakorporale Membranoxygenierung (VV ECMO) ist ein etabliertes Unterstützungsverfahren für Patienten mit sehr schwerem respiratorischem Versagen und konnte während der Coronaviruserkrankung-2019(COVID-19)-Pandemie ihre zentrale Bedeutung für die Behandlung eines sehr schweren Lungenversagens bestätigen. Bakterien und Pilze können zu schweren Infektionen bei kritisch kranken Patienten führen. Diese Arbeit beschreibt das Erregerspektrum bei COVID-19-Patienten mit VV ECMO-Unterstützung in unserem Zentrum.

**Methodik:**

In die retrospektive monozentrische Registeranalyse wurden alle Patienten mit COVID-19-assoziiertem respiratorischem Versagen, die zwischen März 2020 und Mai 2022 mit VV ECMO unterstützt wurden, eingeschlossen. Ergebnisse sämtlicher mikrobiologischer Proben, die im Rahmen der klinischen Routine innerhalb der ersten 30 Tage nach ECMO-Anlage untersucht wurden, wurden erfasst. Die Erreger wurden nach Zeitpunkt, Detektionsort und mikrobiologischen Charakteristika beurteilt.

**Ergebnisse:**

Im Beobachtungszeitraum wurden in unserem Zentrum insgesamt 88 COVID-19-Patienten mit VV ECMO unterstützt. Bei 83/88 Patienten (94,3 %) wurde mindestens ein Erreger nachgewiesen. Die meisten Nachweise erfolgten aus Proben des Respirationstrakts (88,6 %). Der früheste Nachweis gelang aus dem Respirationstrakt nach einer medianen Zeit von 5 Tagen. Die am häufigsten nachgewiesenen Erreger waren *Staphylococcus spp*., *Candida spp.*, *Klebsiella spp.*, *Escherichia coli* und *Enterococcus spp.*

**Schlussfolgerung:**

Bei Patienten mit COVID-19 und VV ECMO-Unterstützung konnten mit einer hohen Frequenz Pathogene nachgewiesen werden.

## Introduction

Venovenous extracorporeal membrane oxygenation (VV ECMO) is an established support option for patients with very severe respiratory failure [4]. Evidence suggests a benefit for appropriately selected patients when supported with ECMO in addition to standard of care including lung-protective invasive mechanical ventilation [6, 7, 11, 13]. During the coronavirus disease 2019 (COVID-19) pandemic, ECMO has played an important role. Increasing experience and evidence helped to optimize patient selection and treatments [19]. In our center, 88 COVID-19 patients were supported with VV ECMO. Overall survival of these patients until day 90 after initiation of ECMO was 50% (44/88). Detailed patient and treatment data have been reported previously [20, 21].

Yet, to date, only little is known about the role of superinfections and nosocomial infections in these severely ill patients [1]. Currently, it remains unclear whether bacterial and fungal co- and superinfections in patients with COVID-19-associated severe respiratory failure are different with respect to frequency, microbiological spectrum, and prognosis, as compared to patients without COVID-19 [9, 10, 14, 16]. Most importantly, evidence is particularly scarce for patients with COVID-19 and ECMO [3, 14, 19]. To shed more light on this issue, we aimed to describe the microbiological spectrum of bacteria and fungi detected in patients with COVID-19-associated respiratory failure supported with VV ECMO in our center.

## Materials and methods

This is a retrospective single-center study. We included all patients with COVID-19-associated severe respiratory failure who were supported with VV ECMO at the Freiburg University Medical Center’s Department for Interdisciplinary Medical Intensive Care between March 18, 2020 and May 15, 2022. Due to the retrospective nature of this study, no patient-related intervention was performed specifically for the purpose of this study, and only information and data collected for routine clinical care of the patients were available. The study was approved by the University of Freiburg’s institutional ethics committee (EK 151/14) and conforms to the ethical guidelines of the 1975 Declaration of Helsinki.

Indication for ECMO followed established criteria in accordance with Extracorporeal Life Support Organization (ELSO) recommendations that were applied unchanged during the entire observation period for all patients included in this analysis [2, 20, 21].

For this analysis, findings from all microbiological blood, sputum, tracheal secretions, bronchial secretions or urine samples that were obtained during clinical routine from the beginning of ECMO support until day 30 after initiation of ECMO were considered. Pathogens were evaluated according to time of sampling (i.e., days after initiation of ECMO), site of detection (i.e., bloodstream, respiratory tract, urinary tract), and microbiological characteristics (i.e., gram-positive or gram-negative bacteria, fungi). If the same pathogen was detected repeatedly from one detection site, only the first detection was included in the analysis.

The following patient demographic and clinical parameters were collected: age, sex, body mass index (BMI), time in the intensive care unit (ICU), duration of ECMO support, 30-day mortality, pre-existing comorbidities, cause of death, and medical therapy for COVID-19.

All data were entered into an electronic chart (Microsoft Excel 2010, Microsoft Corp., Redmond, WA, USA) by members of the study team (C. G., E. W., A. S.) and crosschecked after entry by a second study team member for accuracy. For statistical analyses, Prism (version 9; GraphPad Software Inc., San Diego, CA, USA) was used. Continuous variables were compared using the Mann–Whitney test. *P*-values at or below 0.05 were considered statistically significant.

## Results

At our center, from March 18, 2020 until May 15, 2022, 88 patients with COVID-19-associated respiratory failure received VV ECMO support. Median age (interquartile range, IQR) of the entire cohort was 55.0 (47.3–62.0) years, 28/88 patients (31.8%) were female. Median BMI (IQR) was 30.9 (27.7–36.2) kg/m^2^. Further patient baseline characteristics are displayed in Table 1. Detailed treatment and outcome data have been reported previously [20, 21].

**Table 1:** Baseline and treatment parameters and mortality of patients with COVID-19-associated respiratory failure supported with VV ECMO

	All patients (*n* = 88)	Patients with pathogens isolated from bloodstream samples (*n* = 54)	Patients with pathogens isolated from respiratory tract samples (*n* = 78)	Patients with pathogens isolated from urinary tract samples (*n* = 44)	Patients with pathogen detection in any of the samples (*n* = 83)	Patients without pathogen detection in any sample (*n* = 5)
*Patient characteristics*						
Age [years]	55.0 (47.3–62.0)	56.0 (47.5–63.0)	57.0 (47.0–64.0)	55.0 (46.3–62.8)	55.0 (47.0–63.0)	54.0 (51.5–58.5)
Sex [female]	28 (31.8%)	13 (24.1%)	23 (29.5%)	18 (40.9%)	27 (32.5%)	1 (20.0%)
BMI [kg/m^2^]	30.9 (27.7–36.2)	30.9 (26.6–35.5)	30.9 (27.7–36.3)	30.9 (27.7–35.2)	30.9 (27.7–36.4)	29.4 (26.8–35.7)
Time on ICU [days]	39.0 (21.0–61.0)	42.5 (25.3–64.3)	39.0 (22.0–59.5)	46.0 (29.0–71.0)	41.0 (22.0–62.0)	21.0 (19.0–46.0)
Days on ECMO	21.5 (10.9–39.3)	25.8 (13.5–41.1)	22.7 (11.6–37.8)	32.1 (18.2–49.6)	22.7 (11.2–39.7)	10.9 (3.6–42.2)
30-day mortality	32 (36.3%)	18 (33.3%)	27 (34.6%)	6 (13.6%)	38 (45.8%)	4 (80.0%)
*Comorbidities*						
Diabetes mellitus	18 (20.5%)	9 (16.7%)	17 (21.8%)	3 (6.8%)	17 (20.5%)	1 (20.0%)
Malignancy	3 (3.4%)	2 (3.7%)	3 (3.8%)	1 (2.4%)	0 (0%)	0 (0%)
CHD	8 (9.0%)	6 (11.1%)	8 (10.3%)	4 (9.1%)	0 (0%)	0 (0%)
Arterial hypertension	30 (34.1%)	22 (40.7%)	28 (35.9%)	12 (27.3%)	29 (34.9%)	1 (20.0%)
Immunosuppression	3 (3.4%)	2 (3.7%)	2 (2.6%)	0 (0%)	2 (2.4%)	1 (20.0%)

Results are displayed as *n* (%) or median (IQR)

*COVID-19* coronavirus disease 2019, *VV ECMO* venovenous extracorporeal membrane oxygenation, *BMI* body mass index, *ICU* intensive care unit, *CHD* coronary heart disease, *IQR* interquartile range

**Fig. 1:**
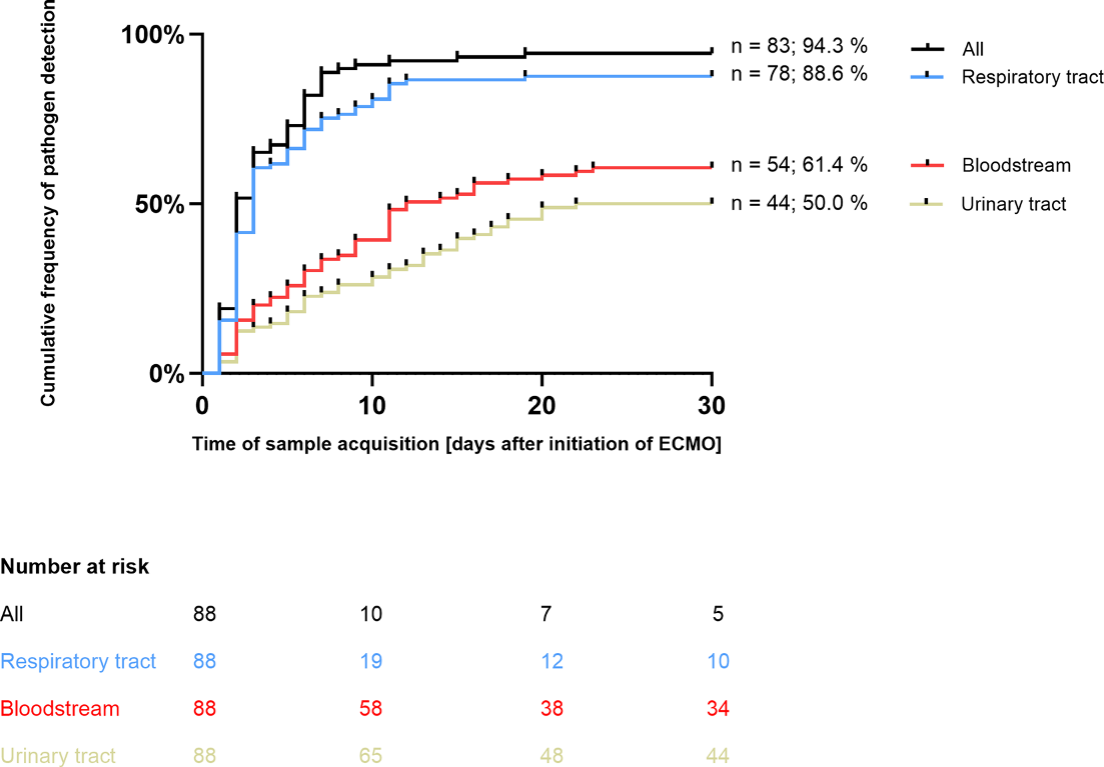
Time of sample acquisition in relation to initiation of extracorporeal membrane oxygenation (*ECMO*; day 0 = start of ECMO) and cumulative frequency of pathogen detection in patients with COVID-19-associated severe respiratory failure during the first 30 days of ECMO support, displayed by site of detection. Cumulative frequency of positive samples mounts within 30 days after initiation of ECMO to 94.3% (all sites combined), 88.6% (respiratory tract), 61.4% (bloodstream), and 50.0% (urinary tract), respectively

**Fig. 2:**
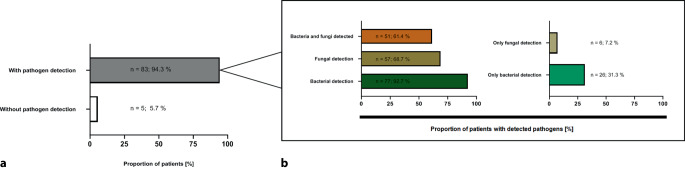
Proportion of patients with and without pathogen detection during the first 30 days after initiation of extracorporeal membrane oxygenation (*ECMO*). **a** In 83/88 patients (94.3%) a pathogen was detected, whereas in 5/88 patients (5.7%) no pathogen was detected. **b** In 77/83 patients (92.7%) bacteria were detected, in 57/83 patients (68.7%) fungi were found. In 51/83 patients (61.4%), both bacteria and fungi were detected, whereas in 6/83 patients (7.2%) only fungi and in 26/83 patients (31.3%) only bacteria were found

**Fig. 3:**
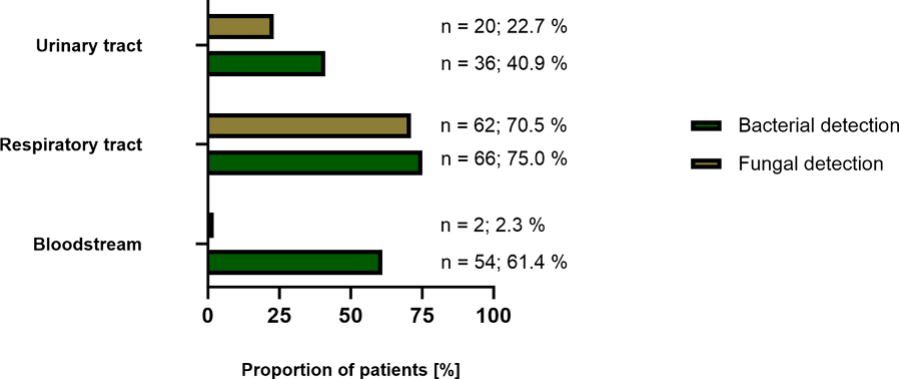
Type of detected pathogens by site of detection. Most pathogens were detected in the respiratory tract; in 66/88 patients (75.0%) bacteria and in 62/88 patients (70.5%) fungi were detected. Bloodstream samples of 54/88 patients (61.4%) were positive for bacteria, but in only 2/88 patients (2.3%) were fungi detected. In urinary tract samples of 36/88 patients (40.9%), bacteria were found and in those of 20/88 patients (22.7%) fungi were detected

In 83/88 patients (94.3%) at least one pathogen was identified in any of the samples analyzed (Fig. 1 and Table 2). In 77/83 patients (92.7%), we found bacteria, and in 57/83 patients (68.7%) fungi were detected (Fig. 2). Until day 30 after initiation of ECMO, pathogens were found in bloodstream samples of 54/88 patients (61.4%), in samples taken from the respiratory tract in 78/88 patients (88.6%), and in samples from the urinary tract in 44/88 patients (50.0%; Fig. 1). Overall, bacteria were detected far more often than fungi (Fig. 3).

**Table 2:** Number of pathogens, causes of death, and concomitant therapy in patients with severe COVID-19 and VV ECMO and additional pathogens detected in the bloodstream, in the respiratory tract or in the urinary tract

	All patients (*n* = 88)	Patients with pathogens isolated from bloodstream samples (*n* = 54)	Patients with pathogens isolated from respiratory tract samples (*n* = 78)	Patients with pathogens isolated from urinary tract samples (*n* = 44)
*Time to detection [days]*	6.0 (2.0–11.7)	8.0 (4.0–13.0)	5.0 (2.0–10.0)	10.0 (5.0–15.0)
*No pathogens detected*	5 (5.7%)	0 (0%)	0 (0%)	0 (0%)
*Number of detected pathogens*				
≥ 1 pathogen	83 (94.3%)	54 (100%)	78 (100%)	44 (100%)
≥ 2 pathogens	73 (82.9%)	28 (51.9%)	57 (73.0%)	22 (50.0%)
≥ 3 pathogens	48 (54.5%)	12 (22.2%)	32 (41.0%)	4 (9.0%)
≥ 4 pathogens	17 (19.3%)	4 (7.4%)	13 (16.7%)	0 (0%)
≥ 5 pathogens	5 (5.6%)	2 (3.7%)	3 (3.8%)	0 (0%)
*Cause of death*				
Sepsis/MOF	15 (17.0%)	11 (20.4%)	13 (16.7%)	4 (9.0%)
Intracerebral bleeding	12 (13.6%)	5 (9.3%)	10 (12.8%)	1 (2.3%)
Respiratory failure	5 (5.7%)	3 (5.6%)	5 (6.4%)	1 (2.3%)
*Concomitant therapy*				
Tocilizumab	28 (31.8%)	20 (37.0%)	24 (30.8%)	18 (40.9%)
Remdesivir	9 (10.2%)	5 (9.3%)	8 (10.3%)	4 (9.0%)
Dexamethasone	75 (85.2%)	46 (85.2%)	67 (85.9%)	40 (90.9%)
Extracorporeal hemoadsorption (Cytosorb)	13 (14.8%)	7 (13.0%)	12 (15.4%)	5 (11.4%)

Results are displayed as *n* (%) or median (IQR)

*COVID-19* coronavirus disease 2019, *VV ECMO* venovenous extracorporeal membrane oxygenation, *MOF* multiple organ failure

### Time of detection

**Fig. 4:**
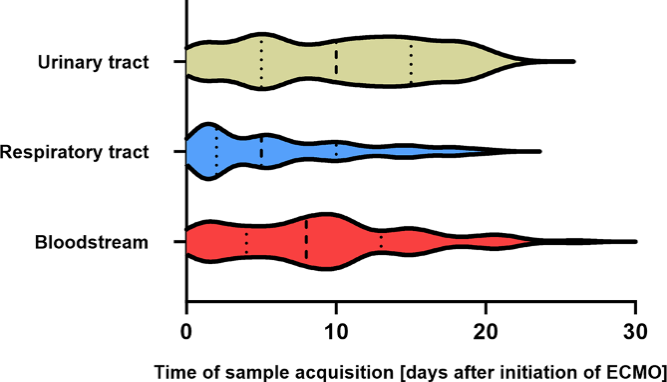
Time to pathogen detection stratified by site of detection in patients with COVID-19-associated respiratory failure during the first 30 days after extracorporeal membrane oxygenation (*ECMO*) initiation. Earliest detection was in samples taken from the respiratory tract with the samples collected at a median time (IQR) of 5.0 (2.0–10.0) days after ECMO initiation, then in the bloodstream after 8.0 (4.0–13.0) days and finally in the urinary tract after 10.0 (5.0–15.0) days

Most pathogens were first detected in samples taken during the first 10 days after initiation of ECMO (Figs. 1 and 4). In only 5 patients, no pathogen was detected after initiation of ECMO. Demographic parameters (i.e., age, sex, BMI) in these patients were comparable to patients who had pathogens detected. Mortality of patients without pathogens was higher in this small subgroup (Table 1).

### Site of detection

Pathogens were detected earliest in samples from the respiratory tract; median duration to first detection (IQR) was 5.0 (2.0–10.0) days, followed by detection in bloodstream samples after a median of 8.0 (4.0–12.8) days. Detection of bacteria and fungi in urine samples occurred later, but frequency of detection increased over time after a median of 10.0 (5.0–15.0) days (Figs. 1 and 4 and Table 2).

### Type of pathogen

**Fig. 5:**
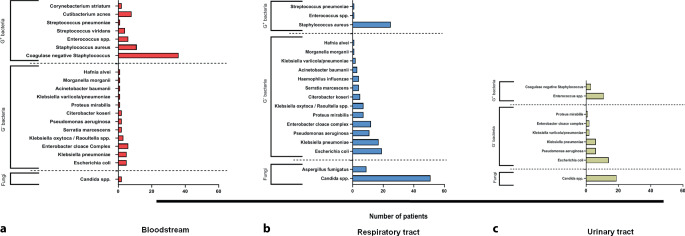
Number of patients for different pathogens detected in samples collected during the first 30 days after initiation of extracorporeal membrane oxygenation (*ECMO*) in patients with COVID-19-associated severe respiratory failure by site of detection (**a–c**)

*Staphylococcus spp*., *Candida spp., Klebsiella spp., Escherichia coli* and *Enterococcus spp.* were the most frequently detected pathogens (Fig. 5). Fungi (*Aspergillus fumigatus, Candida spp.*) were predominantly detected in the respiratory and urinary tract samples and only rarely in blood cultures.

### Time to detection

**Fig. 6:**
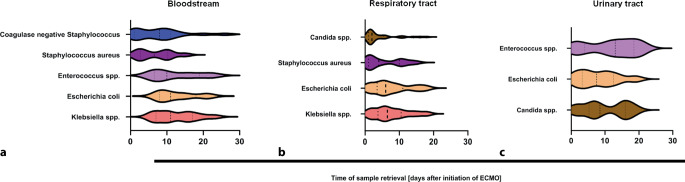
Time to pathogen detection discriminated by the site of detection and specific pathogens in patients with COVID-19-associated respiratory failure during the first 30 days after extracorporeal membrane oxygenation (*ECMO*) initiation. **a** Earliest detection in blood samples was for S*taphylococcus aureus* in samples retrieved at a median time (IQR) of 6.5 (2.8–10.3) days after initiation of ECMO, second were coagulase-negative staphylococci 8.0 (1.0–11.0) days, followed by *Enterococcus spp.* 10.0 (6.5–19.0) days and gram-negative bacteria, such as *Escherichia coli* or *Klebsiella spp.* 11.0 (8.0–18.0) days. **b** *Candida spp.* were found earliest in respiratory tract in samples collected after 2.0 (1.0–5.3) days, then *Staphylococcus aureus* after 4.0 (1.0–10.0) days, later followed by gram-negative bacteria such as *Escherichia coli* after 6.0 (3.5–11.0) days and *Klebsiella spp.* after 6.5 (3.8–10.5) days. **c** In urine samples, *Escherichia coli* was detected first in samples collected after 7.5 (3.3–12.5 days) days, followed by *Candida spp.* after 8.5 (5.3–16.0) days and then gram-positive bacteria like *Enterococcus spp.* after 13.0 (4.0–18.5) days

Time to detection differed between sites of detection and pathogens. In blood cultures, *Staphylococcus aureus* was earliest detected in samples obtained after a median (IQR) of 6.5 (2.8–10.3) days, followed by coagulase-negative staphylococci in samples taken after 8.0 (1.0–11.0) days (Fig. 6 and Table 3). *Enterococcus spp.* were found in samples taken after 10.0 (6.5–19.0) days. Gram-negative bacteria, such as *Escherichia coli* or *Klebsiella spp.,* were detected later, i.e., after 11.0 (8.0–18.0) days and 11.0 (7.0–17.0) days, respectively (Fig. 6 and Table 3).

**Table 3:** Time to pathogen detection [days] separated by pathogen and place of detection

	Bloodstream (*n* = 54)	Respiratory tract (*n* = 78)	Urinary tract (*n* = 44)
Pathogens			
*Staphylococcus aureus*	6.5 (2.8–10.3)	4.0 (1.0–10.0)	N/A
*Coagulase-negative Staphylococcus*	8.0 (1.0–11.0)	N/A	4.0 (1.0–12.0)
*Enterococcus spp.*	10.0 (6.5–19.0)	2.0 (2.0–2.0)	13.0 (4.0–18.5)
*Klebsiella spp.*	11.0 (7.0–17.0)	6.5 (3.8–10.5)	14.0 (9.5–17.3)
*Escherichia coli*	11.0 (8.0–18.0)	6.0 (3.5–11.0)	7.5 (3.3–12.5)
*Candida spp.*	8.0 (2.0–14.0)	2.0 (1.0–5.3)	8.5 (5.3–16.0)

Results are displayed as median (IQR)

*N/A* not available, *spp.* Species, *IQR* interquartile range

In samples taken from the respiratory tract, earliest detection was for *Candida spp.* in samples taken after 2.0 (1.0–5.3) days (Fig. 6 and Table 3). *Staphylococcus aureus* was first detected in samples collected 4.0 (1.0–10.0) days after initiation of ECMO. Gram-negative bacteria prevailed later, e.g., *Escherichia coli* after 6.0 (3.5–11.0) days or *Klebsiella spp.* after 6.5 (3.8–10.5) days (Fig. 6 and Table 3).

*Escherichia coli* was detected earliest in urine samples collected after 7.5 (3.3–12.5) days (Fig. 6 and Table 3). Then, *Candida spp.* were detected in samples after 8.5 (5.3–16.0) days. Gram-positive bacteria, such as *Enterococcus spp.* were observed later 13.0 (4.0–18.5) days (Fig. 6 and Table 3).

## Discussion

We present a comprehensive analysis of microbiological findings in specimens taken during the first 30 days of VV ECMO support from the bloodstream, the respiratory tract, and the urinary tract of patients with COVID-19-associated severe respiratory failure. Bacteria were the most prevalent pathogens, with the majority detected in samples from the respiratory tract.

The frequency of nosocomial infections among patients with COVID-19 varied considerably in previous studies, largely depending on the severity of disease of the patients [5, 8, 12, 18]. A review that included 28 observational studies with more than 5000 patients hospitalized for COVID-19 described secondary pulmonary infections in 16% of the patients, ranging from 4.8–42.8% in the cohorts included in the review, and secondary fungal infections in 6.3% of the patients (range in the reviewed studies between 0.9 and 33.3%) [5].

Overall, the frequency of pathogen detection in our cohort was much higher. In contrast to these previous studies, we merely described microbiological findings from specimens collected during clinical routine, but we did not assess additional criteria of clinically manifest infection. The timing of the collection of samples was not standardized; instead, the initiation of microbial assessment was at the discretion of the treating physicians. This may explain the higher frequency of pathogen detection in our cohort. Furthermore, it is difficult to directly compare different cohorts of ECMO patients due to limited information about potentially relevant secondary diagnoses, comorbidities and additional therapies. Of note, the patients in our cohort were older than the patients in the other large ECMO cohorts described above.

In our cohort, pathogens were detected earliest in the respiratory tract and only little later in the bloodstream and the urinary tract. This observation confirms previous findings from the Italian cohort described above [12]. In this cohort, ventilator-associated pneumonias (VAPs) occurred after a median time of 17 days after hospital admission and 12 days after ICU admission, followed by bloodstream and urinary tract infections on average 19 days after hospital admission (day 14–15 after ICU admission) [12].

The occurrence of secondary infections is not specific to COVID-19 patients. A European multicenter trial involving 1050 patients compared bacterial detection in the first 48 h after intubation in patients with COVID-19 or influenza pneumonia [17]. Similar to our analysis, the majority of detected bacteria were gram-positive, such as *Staphylococcus aureus* or *Streptococcus pneumoniae*, mostly found in samples from the respiratory tract. Nevertheless, the detection period in our cohort was much longer, which possibly explains the higher incidence of coinfections reported here.

In our cohort, various pathogens were isolated from the respiratory tract, the blood and the urinary tract. In samples from the respiratory tract, *Candida spp., Staphylococcus aureus, Escherichia coli* and *Klebsiella pneumoniae *were found most frequently. Detection of *Candida spp.* in this cohort most likely reflects colonization of the respiratory tract without clinical relevance [15]. Comparable to our findings, Grasselli et al. identified gram-negative bacteria most frequently in COVID-19 patients with VAP (64% of the infections), including *Pseudomonas aeruginosa, Enterobacter, Klebsiella species* and *Escherichia coli*. Gram-positive bacteria such as *Staphylococcus aureus* or *Enterococcus species* caused 36% of the infections [12].

In a review of 49 studies, Chong et al. also reported *Pseudomonas aeruginosa, Klebsiella species, Staphylococcus aureus* and *Escherichia coli* as the microorganisms most frequently responsible for secondary respiratory tract infections in critically ill COVID-19 patients [5]. These observations are also well in line with the findings in our cohort. The frequent detection of *Candida spp.* in samples from the urinary tract of patients in our cohort (most of them with indwelling urinary catheters) likely similarly reflects colonization without particular clinical relevance. Bloodstream infections with coagulase-negative Staphylococci or *Staphylococcus aureus* need to be interpreted in the context of various venous and arterial catheters and the ECMO cannulae typically present in these patients.

### Limitations

Several limitations of this study must be mentioned. This is an uncontrolled, retrospective single-center study that reflects the situation at our institution and may not be generalizable to other settings. Due to the retrospective design, no systematic microbiological sampling specifically for the sake of this study was possible. All reported observations must rely on microbiological sampling as indicated during clinical routing and for clinical use. The majority of patients included in this analysis (63/88, 71.6%) were transferred to our center for ECMO support from other hospitals. Information on the treatment of these patients before initiation of ECMO support was limited in many cases. Therefore, we focused our analysis on the first 30 days after initiation of ECMO.

## Conclusion

In this analysis of 88 severely ill COVID-19 patients supported with VV ECMO, we identified bacteria or fungi in samples from the respiratory tract, the blood or the urinary tract in the vast majority of the patients. Further studies should be performed to allow for a more detailed scrutiny of clinical data and detection of pathogens in cohorts of critically ill COVID-19 patients supported with VV ECMO.

## Data Availability

All data will be available from the corresponding author on reasonable request.

## Declarations

Data collection was approved by the institutional ethics committee of the University of Freiburg (EK 151/14); due to the retrospective and observational nature of the study, the need for informed consent was waived.

The supplement containing this article is not sponsored by industry.

## References

[1] Abrams D, Grasselli G, Schmidt M et al (2020) ECLS-associated infections in adults: what we know and what we don’t yet know. Intensive Care Med 46:182–191 31768569 10.1007/s00134-019-05847-z PMC7222121

[2] Badulak J, Antonini MV, Stead CM et al (2021) Extracorporeal Membrane Oxygenation for COVID-19: Updated 2021 Guidelines from the Extracorporeal Life Support Organization. ASAIO J 67:485–495 33657573 10.1097/MAT.0000000000001422 PMC8078022

[3] Barbaro RP, Maclaren G, Boonstra PS et al (2021) Extracorporeal membrane oxygenation for COVID-19: evolving outcomes from the international Extracorporeal Life Support Organization Registry. Lancet 398:1230–1238 34599878 10.1016/S0140-6736(21)01960-7 PMC8480964

[4] Brodie D, Slutsky AS, Combes A (2019) Extracorporeal Life Support for Adults With Respiratory Failure and Related Indications: A Review. JAMA 322:557–568 31408142 10.1001/jama.2019.9302

[5] Chong WH, Saha BK, Ananthakrishnan R et al (2021) State-of-the-art review of secondary pulmonary infections in patients with COVID-19 pneumonia. Infection 49:591–605 33709380 10.1007/s15010-021-01602-z PMC7951131

[6] Combes A, Hajage D, Capellier G et al (2018) Extracorporeal Membrane Oxygenation for Severe Acute Respiratory Distress Syndrome. N Engl J Med 378:1965–1975 29791822 10.1056/NEJMoa1800385

[7] Combes A, Peek GJ, Hajage D et al (2020) ECMO for severe ARDS: systematic review and individual patient data meta-analysis. Intensive Care Med 46:2048–2057 33021684 10.1007/s00134-020-06248-3 PMC7537368

[8] De Hesselle ML, Borgmann S, Rieg S et al (2022) Invasiveness of Ventilation Therapy Is Associated to Prevalence of Secondary Bacterial and Fungal Infections in Critically Ill COVID-19 Patients. J Clin Med 11:10.3390/jcm11175239 PMC9457079 36079168

[9] Fekkar A, Lampros A, Mayaux J et al (2021) Occurrence of Invasive Pulmonary Fungal Infections in Patients with Severe COVID-19 Admitted to the ICU. Am J Respir Crit Care Med 203:307–317 33480831 10.1164/rccm.202009-3400OC PMC7874326

[10] Garcia-Vidal C, Sanjuan G, Moreno-Garcia E et al (2021) Incidence of co-infections and superinfections in hospitalized patients with COVID-19: a retrospective cohort study. Clin Microbiol Infect 27:83–88 32745596 10.1016/j.cmi.2020.07.041 PMC7836762

[11] Goligher EC, Tomlinson G, Hajage D et al (2018) Extracorporeal Membrane Oxygenation for Severe Acute Respiratory Distress Syndrome and Posterior Probability of Mortality Benefit in a Post Hoc Bayesian Analysis of a Randomized Clinical Trial. JAMA 320:2251–2259 30347031 10.1001/jama.2018.14276

[12] Grasselli G, Scaravilli V, Mangioni D et al (2021) Hospital-Acquired Infections in Critically Ill Patients With COVID-19. Chest 160:454–465 33857475 10.1016/j.chest.2021.04.002 PMC8056844

[13] Munshi L, Walkey A, Goligher E et al (2019) Venovenous extracorporeal membrane oxygenation for acute respiratory distress syndrome: a systematic review and meta-analysis. Lancet Respir Med 7:163–172 30642776 10.1016/S2213-2600(18)30452-1

[14] Network C‑IGOBOTR, C‑ICUI (2021) Clinical characteristics and day-90 outcomes of 4244 critically ill adults with COVID-19: a prospective cohort study. Intensive Care Med 47:60–73 33211135 10.1007/s00134-020-06294-x PMC7674575

[15] Pappas PG, Kauffman CA, Andes DR et al (2016) Executive Summary: Clinical Practice Guideline for the Management of Candidiasis: 2016 Update by the Infectious Diseases Society of America. Clin Infect Dis 62:409–417 26810419 10.1093/cid/civ1194

[16] Pickens CO, Gao CA, Cuttica MJ et al (2021) Bacterial Superinfection Pneumonia in Patients Mechanically Ventilated for COVID-19 Pneumonia. Am J Respir Crit Care Med 204:921–932 34409924 10.1164/rccm.202106-1354OC PMC8534629

[17] Rouze A, Martin-Loeches I, Povoa P et al (2021) Early Bacterial Identification among Intubated Patients with COVID-19 or Influenza Pneumonia: A European Multicenter Comparative Clinical Trial. Am J Respir Crit Care Med 204:546–556 34038699 10.1164/rccm.202101-0030OC PMC8491267

[18] Sang L, Xi Y, Lin Z et al (2021) Secondary infection in severe and critical COVID-19 patients in China: a multicenter retrospective study. Ann Palliat Med 10:8557–8570 34379989 10.21037/apm-21-833

[19] Supady A, Combes A, Barbaro RP et al (2022) Respiratory indications for ECMO: focus on COVID-19. Intensive Care Med 48:1326–1337 35945343 10.1007/s00134-022-06815-w PMC9362963

[20] Widmeier E, Wengenmayer T, Maier S et al (2022) Extracorporeal membrane oxygenation during the coronavirus disease 2019 pandemic: Continued observations from a retrospective single-center registry. Artif Organs 46:2329–2333 35857712 10.1111/aor.14365 PMC9349474

[21] Widmeier E, Wengenmayer T, Maier S et al (2022) Extracorporeal membrane oxygenation during the first three waves of the coronavirus disease 2019 pandemic: A retrospective single-center registry study. Artif Organs 46:1876–1885 35451145 10.1111/aor.14270 PMC9111358

